# Factors Influencing the Quality of Life of Healthy Pregnant Women in North Jordan

**DOI:** 10.3390/medicina55060278

**Published:** 2019-06-15

**Authors:** Ghadeer Alzboon, Gülşen Vural

**Affiliations:** Department of Birth and Women’s Health Nursing, Near East University, 99138 Nicosia, Cyprus, via Mersin 10, Turkey; gulsenural@hotmail.com

**Keywords:** healthy pregnancy, quality of life, perceived stress, perceived social support

## Abstract

*Background and Objectives*: Quality of life (QOL) assessment during pregnancy contributes to determining women’s unmet needs and preventing negative health outcomes. In this study, we aimed to identify the effects of participants’ characteristics, perceived stress, and perceived social support on their QOL. We also aimed to determine the differences in QOL according to these factors. *Materials and Methods*: A cross-sectional study was carried out in a city in Jordan. Purposive sampling was used to select 218 participants. Data was collected by the quality of life Short Form- 36(SF-36) survey, perceived stress scale (PSS), and The Multidimensional perceived Social Support Scale (MSPSS). *Results*: We found that only parity had a significant effect on the QOL. High-parity women had lower QOL scores than low-parity women. The participants reported high social support, specifically from their families and significant others. The 36-Item Short-Form Health Survey was a reliable tool for measuring the QOL in pregnancy. *Conclusions*: Parity factor and social support should be recognized in any health promotion intervention and during providing antenatal care. Further research is needed toassess the QOL during pregnancy.

## 1. Introduction

Quality of life (QOL) assessment has recently attracted growing interest in health care research and practice as a means to promote health and prevent illness. The World Health Organization defined QOL as an “individual’s perception of their physical health, psychological state, level of independence, social relationships, personal beliefs, and their relationships to silent features of their environment” [1]. In the context of pregnancy, measuring women’s perceived QOL and the factors influencing it can help in identifying unmet needs and predicting future health problems.

Pregnancy is not a pathological condition [2]; it includes physiological, anatomical, and biochemical changes in the woman’s body [3]. These normal changes and their interactions with certain factors (e.g., economic factors and stressful events) may adversely affect women’s health throughout the stages of pregnancy even if they do not have any medical or psychological disease. This consequently affects women’s usual activities and QOL [4]. The literature has shown that poor QOL in pregnancy is associated with negative health consequences. For example, the possibility of having low birth-weight infants increases among pregnant women with low QOL [5,6]. Additionally, low QOL in pregnancy contributed to low QOL in the postnatal period [7] and higher gestational weight gain [8]. Moreover, low QOL was associated with the experience of pregnancy related symptoms such as fatigue, back pain, and pelvic pain [9]. 

Researchers have investigated the relationship between QOL and various factors during pregnancy, such as socio-demographic and obstetric characteristics, perceived social support, and perceived stress. The reported QOL scores were low in women of young age [10] and low socioeconomic status (low income and low educational level) [11]. Low socioeconomic status has been associated with late antenatal care [12] and women’s dissatisfaction with life [13], which leads to poor health and QOL [14,15].

Studies on the obstetric factors showed that low-parity pregnant women had the highest QOL scores [11,16,17]. Researchers have posited that low-parity women have a positive psychological reaction toward pregnancy in contrast to high-parity women who have multiple roles and duties. The lowest QOL scores were reported in the third trimester of pregnancy, and the highest scores were reported in the second trimester [6,18]. Women in late pregnancy may have some physical (e.g., back pain and insomnia) and psychological discomforts (e.g., fear of birth and worries about the baby’s health), which indicate poor QOL. 

Unplanned pregnancy has been associated with low QOL, poor self-care behavior, and less antenatal care [19]. Moreover, women with unplanned pregnancies had a poor-quality relationship with partners, and a low level of social support [20]. Lack of social support was associated with poor QOL [21], postpartum depression, and a high level of perceived stress [22].

Perceived stress is an estimate of women’s feelings and thoughts about stressful events and the ability to cope with the experienced stress [23]. Most of the reviewed literature hypothesized that the QOL was a determinant of perceived stress [15,24,25]. But, the study of Lau et al. [15] indicates that the direction of causality between the two variables is not clear. Women of high perceived stress scores may engage in unhealthy behavior such as smoking [26] and may have sleep disturbances [27]. Consequently, health problems may be developed which may lead to adverse physical and mental health outcomes. Thus, our study investigated the effect of perceived stress on the QOL during pregnancy. Theoretically, psychological health declines in pregnancy, especially among women of low socioeconomic status [28,29], those with unplanned pregnancies [30], and those who lack social support [31,32,33]. In an Iranian study, poor QOL was associated with a high stress level and less social support among healthy pregnant women in the first and second trimesters [25].

The main factors associated with high QOL in pregnancy were assessed in one systematic review [34]. These factors were the mean maternal age, a high educational level, high income, being primiparous, being in the first trimester of pregnancy, having positive feelings toward pregnancy, high social support from family and friends, low stress and anxiety, and doing physical exercise. Most of the reviewed studies were conducted among pregnant women with pathological conditions and did not include all stages of pregnancy. Nineteen studies used the 36-Item Short-Form Health Survey (SF-36).

The reviewed literature showed the gap in knowledge about the factors that most influence QOL in pregnancy in some countries. In Jordan, to our knowledge, no empirical studies have investigated the factors related to QOL in pregnancy. In addition, no studies have used the SF-36 in the context of pregnancy; the SF-36 has only been used among the adult population and women of reproductive age in Jordan.

Therefore, in this study, we aimed to identify the factors that affect QOL among healthy pregnant women in north Jordan. We aimed to examine the differences in QOL according to the socio-demographic and obstetric factors, perceived stress, and perceived social support of pregnant women. Identifying the characteristics of pregnant women with poor QOL contributes to increasing the knowledge of health care professionals (e.g., obstetricians, nurses, and midwives) and helps them provide efficient, adequate, and holistic care for pregnant women with poor QOL. The results could be a valuable guide in designing effective health promotion and education programs that focus on improving maternal health and decreasing maternal morbidity and mortality.

## 2. Methods

### 2.1. Study Design and Participants

This cross-sectional study was conducted among healthy pregnant women from June to October 2018. The participants were recruited from those seeking antenatal care in government maternal health settings in a city in Jordan. The participants answered the questionnaire while waiting for antenatal care. Completing the questionnaire took 20 to 25 min.

Purposive sampling was used to get more variation in the sample characteristics. The sample size was estimated according to the rule of thumb of 15 to 20 participants per variable for regression analysis [35]. The estimated sample size was 180 to 200. The sample was increased to 230 to get more variation in the participants’ characteristics. Seven pregnant women refused to participate in the study because they did not stay in the waiting area after registration and were busy with other activities around the hospital. Five unanswered questionnaires were not included in the study. Thus, the final sample size was 218.

The women who were included in the study were married pregnant Jordanian women who could speak and write in Arabic, were carrying a single fetus, and conceived naturally. The exclusion criteria were as follows: divorced and widowed pregnant women (because they were most likely in a negative psychological state that would influence the perceived stress score), women with previous or current adverse obstetric disorders (e.g., preeclampsia, recurrent miscarriages, and preterm labor), women with medical disorders (e.g., cardiac disease, diabetes mellitus, and anemia), women with mental or psychiatric disease (those with any psychiatric problem or those receiving any psychiatric medication), smokers, and women who were taking any medication aside from pregnancy vitamins. The primary investigator and one antenatal staff member (nurse or midwife) identified the women who met the inclusion criteria after the registration process in the antenatal setting.

### 2.2. Measures

#### 2.2.1. Socio-Demographic and Obstetric Questionnaire

The questionnaire contained items about the maternal age, level of education, total monthly income, occupation, parity (number of children), gestational age, and planning for pregnancy.

#### 2.2.2. The SF-36

As part of the RAND Medical Outcomes Study, Ware and Sherbourne developed the SF-36 to evaluate the perceived QOL in the areas of physical and mental health [36,37]. The SF-36 includes eight subscales: physical functioning, role limitations due to physical health problems, role limitations due to emotional problems, social functioning, general mental health (psychological distress and psychological well-being), bodily pain, vitality (energy/fatigue), and general health perception. The subscale scores range from 0 to 100, with 0 representing the worst state of health and 100 indicating the best state of health [36,38]. The total scores were converted to percentiles and classified as low (0–33), moderate (33.4–66.6), or high (66.7–100).

The internal reliability (alpha coefficients) of the SF-36 ranged from 0.73 to 0.81 [36]. The reliability of the Arabic version of the SF-36 among Saudi Arabian citizens, which was measured by the Pearson correlation coefficient, ranged from 0.73 to 0.92 [39]. The alpha coefficient in a study conducted among Jordanian nurses was 0.92 [40]. In the present study, the internal consistency was 0.86.

#### 2.2.3. The Perceived Stress Scale (PSS)

The PSS was developed by Cohen et al. in 1983 to measure the degree to which the respondent perceives the life situations as stressful [41]. The scale is composed of 10 questions on a 5-point Likert scale ranging from 0 (never) to 4 (very often). The total score ranges from 0 to 40, with 40 representing the highest level of perceived stress and 20 indicating a medium level of perceived stress. The PSS is a reliable and valid measure of stress among the Jordanian population. The reliability coefficient of the Arabic version of the PSS among Jordanian students and pregnant women was 0.74 [42]. The reliability coefficient in the present study was 0.68.

#### 2.2.4. The MSPSS

The MSPSS measures individuals’ perceptions of social support from family, friends, and significant others [43]. The MSPSS comprises 12 items on a 7-point Likert scale ranging from 1 (very strongly disagree) to 7 (very strongly agree). The total score ranges from 12 to 84. The Arabic version of the MSPSS was used to assess the perceived social support among Jordanian parents [32] and Jordanian women in the postpartum period [44]; the reliability coefficient of the Arabic MSPSS was 0.87. In the current study, the reliability coefficient of the MSPSS was 0.90.

### 2.3. Ethical Considerations

We conducted this study after obtaining ethical approval from the Jordanian Ministry of Health (Code No. MOH REC 180014, approved on 29.01.2018.) and the Institutional Review Board of the Near East University (Code NO. YDU/2018/56-538, approved onN29.03.2018). The women answered the questionnaire in a private room in the health care setting. Informed consent was obtained from the pregnant women who participated in the study. After we collected the data, we separated the consent form from the questionnaire and kept it in an envelope to ensure data privacy and confidentiality. The participants did not receive any incentives.

### 2.4. Data Analysis

Data were analyzed using SPSS version 22.0. Descriptive statistics were used to describe the participants’ characteristics (frequencies and percentages) and to determine the level of perceived QOL, perceived stress, and perceived social support (means and standard deviations). One-way analysis of variance (ANOVA) was used to examine the differences in the QOL scores according to the investigated factors. The effects of the investigated factors on the QOL were determined using linear regression analysis followed by stepwise regression analysis. The QOL was the dependent variable, and the other factors were the independent variables. The level of significance in this study was *p*<0.05.

## 3. Results

Table 1 shows the socio-demographic and obstetric characteristics of the sample. The proportion of women who did not have a child (parity = 0) was 27.5%, and the proportion of high-parity women (those with four or more children) was 17.4%. About 25% of the women were in the first trimester of pregnancy, 36.2% were in the second trimester, and 38.1% were in the third trimester. The proportion of planned pregnancy (51.8%) was slightly higher than that of unplanned pregnancy (48.2%).

Table 2 shows that the women had moderate QOL (M = 51.85, SD = 14.705). The subscale with the lowest mean score was that of role limitations due to physical health problems (M = 37.16, SD = 36.259), whereas general health had the highest mean score (M = 59.77, SD = 14.542). The total mean scores of the PSS and MSPSS were 20.26 (SD = 5.697) and 63.78 (SD = 13.816), respectively. This indicates that the women had moderate stress and high social support. For the MSPSS subscales, the friends subscale had the lowest score (M = 18.18, SD = 5.964), whereas the significant other (M = 23.39, SD = 5.164) and family subscales (M = 22.2, SD = 5.488) had the highest mean scores.

Statistically significant differences in the QOL scores were found between the parity groups (F = 2.413, *p* = 0.05; see Table 3). The Scheffe post hoc test was applied (Table 4). High-parity women had significantly lower QOL than low-parity women (those with no children or just one child). Although there were no differences in the QOL according to gestational age, women in the third trimester of pregnancy had the lowest mean QOL score (M = 49.91, SD = 15.328), while those in the second trimester had the highest mean QOL score (M = 53.37, SD = 13.7).

Linear regression analysis was employed to determine the effects of the investigated factors on the QOL. This was followed by stepwise regression analysis. In the linear regression analysis (Table 5), the investigated factors explained 6% of the variance in the QOL. None of the independent variables were related to the QOL (F = 1.464, *p* = 0.163). Table 6 shows that only parity predicted the QOL of pregnant women (β = −0.192, F = 8.274, *p* = 0.004).

## 4. Discussion

In this study, we investigated the factors influencing the QOL of healthy pregnant women in Jordan. The pregnant women in our study had moderate QOL scores (M = 51.85). This result is consistent with the results for United States pregnant women (M = 49.37–51.14) [8] and Jordanian adult women (M = 52) [46]. We found that low proportions of women had teenage pregnancies, completed primary school only, completed graduate education, and had a high income. These findings are consistent with the statistics for Jordanian women. The national teenage pregnancy rate was 5% [47]. The illiteracy rate was 7.5% and the unemployment rate was 31.2% among Jordanian women [48]. Women with a high educational level, those who are employed [49], and those with high incomes are most likely to receive maternal care from private rather than public settings.

Our results for parity were consistent with those of other studies [11,16,17,50]. Low-parity women had higher QOL scores than high-parity women. Increased household duties and child-rearing tasks among high-parity women negatively affect their physical and mental health [51]. High-parity women are also more likely to have a decreased happiness level [52] and to be dissatisfied with their lives [13].

High-parity women use antenatal health services less frequently than low-parity women do [53,54]. Women’s increased responsibilities and delay in seeking care contribute to poor QOL. However, our results did not confirm the impact of pregnancy on QOL. Thus, the differences in QOL between pregnant and non-pregnant women need to be examined further.

Our findings for gestational age were also consistent with those of other studies [6,16]. There was no difference in the QOL scores between women in different trimesters of pregnancy. However, as other studies have found [18,55], women in the third trimester of pregnancy had the lowest mean QOL score, specifically for role limitations due to physical problems. These differences in QOL between women in different trimesters of pregnancy are linked to pregnancy-related symptoms and discomforts. For example, women experience nausea and vomiting in the first trimester [17,56] and urinary incontinence in the third trimester [57]. A longitudinal study can be conducted to assess the impact of these discomforts on the QOL throughout pregnancy.

We found no significant difference in the QOL between women with planned and unplanned pregnancies. Other studies had similar findings [14,58]. Women with unplanned pregnancies do not necessarily have ambivalent or negative feelings toward pregnancy. They may accept the reality of being pregnant, cope positively, and be happy, specifically when they receive strong social support, as our results showed.

Social support and perceived stress did not affect the QOL of pregnant women. By contrast, studies have revealed that low QOL in pregnancy was associated with high perceived stress [15,24,25] and low social support [22]. The mean scores for perceived stress and social support in our study were consistent with the scores for Jordanian women. The total mean for the MSPSS was 58.9 (SD = 15.1). The highest mean scores were for the family and significant other subscales. The total mean for the PSS was 27.0 (SD = 9.33) [32]. In Jordan, pregnancy is generally viewed as a positive life event, and families have a strong bond and good relationships. Consequently, Jordanian women and their families have positive feelings toward pregnancy, especially when the pregnancy is planned. However, the experience of normal body changes in pregnancy may cause psychological discomforts and stress. Social support as an emotional coping mechanism plays a significant role in buffering the negative effect of stress [59]. Positive coping strategies such as positive healthy behavior are associated with good QOL [60]. In this study, women’s feelings at the time of data collection may not reflect their actual perceived stress and QOL related to pregnancy.

Great care should be taken in assessing the QOL and its influencing factors during pregnancy in maternal care settings. Health care professionals should recognize the differences between pregnant women, specifically in terms of parity, when providing antenatal care and planning health education and promotion programs. For example, in community-based interventions that cover pregnant women and their social support systems, husbands and families should be involved in antenatal care to improve the QOL of pregnant women.

### Strengths and Limitations

Despite the non-significant effect of perceived stress on the QOL, this is may be the first study that addressed if perceived stress may predate QOL during pregnancy. Moreover, our study supports previous empirical evidence regarding the reliability of the SF-36 in measuring QOL during pregnancy. However, the findings of this study must be interpreted in the light of some limitations. First, data were collected quantitatively at a single point in time using a cross-sectional design. To overcome this limitation, two further research methodologies were recommended to be employed; qualitative and longitudinal design. A qualitative approach should be implemented to assure the quality of data in present study. And a longitudinal design could reveal the changes in women’s perceptions of QOL throughout the pregnancy. Second, limited factors were investigated for their influence on QOL. Future research in needed to investigate life style factors, obesity, and violence behavior. Finally, the private health care settings were not included in sampling procedure. The study was carried out in public health care settings only, which might limit the generalizability of the study findings. Despite these limitations, however, this preliminary investigation could be useful for planning future QOL studies in context of pregnancy.

## 5. Conclusions

QOL assessment during pregnancy is essential in health care. Determining the factors influencing QOL is crucial. In this study, only parity influenced the QOL of pregnant women. High-parity women had a lower QOL than low-parity women. Health care professionals should provide specific care for high-parity women in maternal care settings, such as including women’s husbands and family in antenatal classes and social support interventions. This study is a preliminary assessment of the QOL among Jordanian pregnant women. Further research is needed to address other determinants of QOL using different research methodologies.

## Figures and Tables

**Table 1:** Characteristics of the pregnant women (*n* = 218).

Variables	Frequency	%
Age		
less than19 years old	10	4.6
20–25	61	28.0
26–30	68	31.2
31–35	43	19.7
36 or more	36	16.5
Educational level		
Primary school	12	5.5
Secondary school	73	33.5
College diploma	45	20.6
Bachelor’s degree	79	36.2
Graduate degree	9	4.1
Occupation		
House wife	162	74.3
Part-time worker	21	9.6
Full-time worker	35	16.1
Total monthly family income		
Less than JD ^1^ 450	110	50.5
JD 450–800	80	36.7
More than JD 800	28	12.8
Parity ^2^		
0	60	27.5
1	40	18.3
2	46	21.1
3	34	15.6
4 or more	38	17.4
Gestational age		
1st trimester	56	25.7
2nd trimester	79	36.2
3rd trimester	83	38.1
Planned pregnancy		
Yes	113	51.8
No	105	48.2
Total	218	100.0

^1^ Jordan dinar, JD 1 = USD 1.41.^2^ Parity: “the number of children previously borne” (“Parity”, n.d.) [45].

**Table 2:** Mean scores for quality of life, perceived stress, and perceived social support among healthy pregnant women.

Variable	Mean	Standard Deviation
Quality of life subscales		
Physical functioning	57.80	22.733
Role limitations due to physical health problems	37.16	36.259
Role limitations due to personal or emotional problems	42.97	41.031
Energy/fatigue	42.45	17.833
General mental health	52.68	19.855
Social functioning	58.89	22.463
Pain	54.90	24.386
General health	59.77	14.542
Total quality of life	51.85	14.705
PSS^1^	20.26	5.697
Total MSPSS^2^	63.78	13.816
Significant other	23.39	5.164
Family	22.20	5.488
Friends	18.18	5.964

^1^ PSS: Perceived stress scale scores. ^2^ MSPSS: Multidimensional scale of perceived social.

**Table 3:** Means, standard deviations, and one-way ANOVA of quality of life according to parity.

Variable	N	Mean	Standard Deviation	F	*p*-Value ^1^
Parity					
0	60	54.52	13.968	2.413	0.050
1	40	54.41	13.251		
2	46	51.36	14.993		
3	34	51.40	16.265		
4 or more	38	45.95	14.409		

^1^ The *p*-values were calculated using ANOVA.

**Table 4:** ANOVA comparisons of quality of life according to parity using the Scheffe post hoc test.

Parity(I)	Parity(J)	Mean Difference (I−J)	Standard Error	*p*-Value
0	1	0.11	2.963	0.970
	2	3.16	2.845	0.268
	3	3.12	3.116	0.318
	4 or more	8.58 *	3.010	0.005
1	0	−0.11	2.963	0.970
	2	3.05	3.138	0.332
	3	3.01	3.386	0.375
	4 or more	8.47 *	3.289	0.011
2	0	−3.16	2.845	0.268
	1	−3.05	3.138	0.332
	3	−0.04	3.283	0.990
	4 or more	5.42	3.182	0.090
3	0	−3.12	3.116	0.318
	1	−3.01	3.386	0.375
	2	0.04	3.283	0.990
	4 or more	5.45	3.427	0.113
4 or more	0	−8.58 *	3.010	0.005
	1	−8.47 *	3.289	0.011
	2	−5.42	3.182	0.090
	3	−5.45	3.427	0.113

* The mean difference is significant at *p* = 0.05.

**Table 5:** Linear regression results for factors influencing the quality of life.

	Standardized Beta Coefficients	t	Sig.	R	R^2^	F	*p*-Value ^1^
Age	0.022	0.234	0.815	0.244	0.060	1.463	0.164
Educational level	0.044	0.569	0.570	-	-	-	-
Total monthly family income	−0.068	−0.914	0.362	-	-	-	-
Occupation	−0.020	−0.258	0.796	-	-	-	-
Parity	−0.182	−0.936	0.350	-	-	-	-
Gestational age	−0.091	−1.316	0.190	-	-	-	-
Planned pregnancy	0.060	0.872	0.384	-	-	-	-
PSS ^2^	−0.054	−0.777	0.438	-	-	-	-
MSPSS ^3^	0.083	1.205	0.229	-	-	-	-

^1^ The *p*-value was calculated using regression analysis. ^2^ PSS: Perceived stress scale scores. ^3^ MSPSS: Multidimensional scale of perceived social.

**Table 6:** Stepwise regression results for quality of life according to parity.

	Standardized Beta Coefficient	t	R	R^2^	F	*p*-Value ^1^
Parity	−0.192	−2.876	0.192	0.037	8.274	0.004

^1^ The *p*-value was calculated using stepwise regression analysis. Dependent variable: Quality of life.
